# Clinical development landscape of CAR/TCR-engineered cellular therapies in musculoskeletal sarcomas: a TrialTrove-based registry analysis

**DOI:** 10.3389/fimmu.2026.1939952

**Published:** 2026-10-28

**Authors:** Xiaodong Jia, Qingyu Chen, Yaoyi Wang, Yemeng He, Jinhang Wang, Mingyue Yan

**Affiliations:** 1 Knee Preservation Center, The Affiliated Hospital of Qingdao University, Qingdao, Shandong, China; 2 Suzhou Stomatological Hospital, Suzhou, China; 3 The Second School of Medicine, Wenzhou Medical University, Wenzhou, Zhejiang, China; 4 School of Laboratory Medicine and Life Sciences, Wenzhou Medical University, Wenzhou, Zhejiang, China; 5 Renji College of Wenzhou Medical University, Wenzhou, Zhejiang, China

**Keywords:** basket trial, car-t, clinical trial landscape, engineered cellular therapy, musculoskeletal sarcoma, TCR-T, TrialTrove

## Abstract

**Background:**

CAR/TCR-engineered cellular therapies are increasingly investigated in musculoskeletal sarcomas, but the clinical development portfolio spans heterogeneous histologies, platforms, antigens, and basket protocols. We mapped the TrialTrove-derived landscape while explicitly separating engineered-receptor products from other cell-based immunotherapies.

**Methods:**

TrialTrove clinical trial data were retrieved on 10 June 2026 and constituted the sole data source. Candidate records were identified using prespecified musculoskeletal-sarcoma disease terms and CAR/TCR-engineered-cell product terms. Two investigators independently assessed eligibility and assigned standardized classifications, with disagreements adjudicated by a third investigator. Potential duplicate representations were evaluated using TrialTrove, NCT, and normalized secondary identifiers plus study-level characteristics. We summarized temporal, geographic, phase, status, sponsor, protocol-planned enrollment, platform, target, disease-scope, co-intervention, concentration, and field-availability characteristics. Broader solid-tumor records with explicit sarcoma eligibility were reported separately, the export did not permit consistent determination of actual sarcoma enrollment or a dedicated sarcoma cohort.

**Results:**

Of 140 identified records, 121 were eligible: 38 (31.4%) were sarcoma-specific and 83 (68.6%) were broader solid-tumour records with explicit sarcoma eligibility. Among 110 records with a start year, 63 (57.3%) began during 2021-2026. Phase I or I/II accounted for 108/121 records (89.3%), and median protocol-planned enrollment was 24 participants (IQR 15-49, n=103). The United States and China contributed 55.2% of 163 trial-country links. TCR-T and CAR-T accounted for 60 (49.6%) and 52 (43.0%) records, respectively. NY-ESO-1 contributed 45/125 trial-target links (36.0%), after excluding 12 Unspecified reporting links, the three most frequent specified antigens contributed 66/113 specified-antigen links (58.4%).

**Conclusions:**

Engineered cellular-therapy development in musculoskeletal sarcomas has accelerated but remains predominantly early phase, geographically and antigenically concentrated, and represented largely by broader solid-tumour protocols that list sarcoma eligibility. Prespecified sarcoma strata, dedicated and independently analyzable cohorts, biomarker enrichment, harmonized outcomes, and transparent adjunct-strategy reporting should strengthen future evidence generation. This TrialTrove-based record analysis does not establish actual sarcoma enrollment in basket protocols, comparative efficacy, clinical superiority of a platform or antigen, or exhaustive global coverage.

## 1 Introduction

Musculoskeletal sarcomas are rare malignant mesenchymal tumors arising in bone or soft tissue and encompass numerous histological and molecular entities across the age spectrum (1). Their clinical behavior, treatment sensitivity, and age distribution vary substantially among osteosarcoma, Ewing sarcoma, synovial sarcoma, rhabdomyosarcoma, and the many soft-tissue sarcoma subtypes. For localized disease, multidisciplinary management centered on surgery, radiotherapy when indicated, and histology-adapted systemic therapy remains fundamental, treatment selection also depends on molecular features, disease extent, resectability, and prior therapy (2, 3). Despite advances in diagnosis and local control, recurrent, unresectable, or metastatic sarcoma frequently leaves patients with limited prospects for durable disease control and a continuing need for new systemic strategies (4). Rarity and biological diversity also fragment eligible populations across subtypes and centers. These features create a rationale for antigen-directed cellular therapies, while requiring development programs that preserve disease-specific interpretation rather than treating sarcoma eligibility as a biologically uniform category.

Engineered cellular therapies provide two complementary routes to antigen-directed treatment. Chimeric antigen receptor (CAR) cells use a synthetic receptor that typically combines an extracellular antigen-binding domain, a hinge and transmembrane region, and intracellular activation and costimulatory modules, they recognize cell-surface antigens independently of human leukocyte antigen (HLA) presentation. T-cell receptor-engineered T cells (TCR-T) instead express transferred TCR α/β chains that recognize intracellularly derived peptides presented by defined HLA molecules (5). CAR engineering therefore broadens HLA-independent surface targeting, whereas TCR engineering extends the addressable antigen space to intracellular proteins but narrows eligibility through peptide specificity and HLA restriction. Neither route overcomes by itself the antigen heterogeneity, on-target/off-tumor toxicity, trafficking, persistence, dysfunction, and immunosuppressive microenvironment that constrain solid-tumor therapy (8–10). A platform or target appearing in a trial registry consequently indicates development activity, not established biological validity, therapeutic activity, or clinical readiness.

Therapeutic products are commonly generated by isolating and activating autologous or donor-derived cells, introducing a receptor with integrating viral vectors or non-viral systems such as transposons, messenger RNA, or targeted gene editing, expanding the cells, and completing release testing before infusion. Autologous manufacturing limits donor alloreactivity but is patient-specific and can be vulnerable to collection quality, production time, and manufacturing failure (6, 7). Allogeneic products could support more standardized and immediately available manufacture, but may require disruption of endogenous TCR or HLA pathways and must control graft-versus-host disease, host rejection, and prolonged immunosuppression. Engineering choices also introduce platform-specific risks, including insertional events, off-target editing, TCR chain mispairing or cross-reactivity, and uncontrolled activity, suicide switches or other pharmacological control elements can provide an additional safety layer.

Sarcoma has nevertheless supplied important clinical precedents for engineered T-cell therapy in solid tumors. Early treatment with NY-ESO-1-reactive TCR-engineered lymphocytes produced antitumor responses in selected patients with metastatic synovial sarcoma (11), and subsequent follow-up linked clinical activity with the expansion and persistence of transferred cells (12). A separate phase I/II study in Japan further examined an HLA-A*02-restricted, NY-ESO-1-directed product in biomarker-selected synovial sarcoma (13). Development has also extended to myxoid/round cell liposarcoma through evaluation of letetresgene autoleucel, another NY-ESO-1-directed TCR therapy (14). For MAGE-A4, afamitresgene autoleucel was assessed first in a phase I study that included multiple tumor types and subsequently in the international phase II SPEARHEAD-1 trial in synovial sarcoma (15, 16). In 2024, the product received US regulatory approval for adults with unresectable or metastatic synovial sarcoma after prior chemotherapy whose tumors express MAGE-A4 and who carry specified HLA-A*02 alleles, illustrating both therapeutic progress and tightly bounded eligibility (17). CAR programs have pursued surface antigens in parallel. B7-H3 expression has been documented across examined soft-tissue sarcoma specimens, and preclinical work established a rationale for B7-H3-directed CAR-T cells in pediatric solid-tumor models, including osteosarcoma and Ewing sarcoma (18, 19). Early systemic B7-H3 CAR-cell studies have since tested feasibility across relapsed or refractory solid tumors, although the enrolled disease mix and product design differ between programs (20, 21). GD2-directed CAR-T therapy has also undergone phase I evaluation in children and young adults with osteosarcoma or neuroblastoma, providing evidence of feasibility and cellular expansion rather than a general efficacy conclusion (22). Collectively, these studies show a field advancing through selected antigen, HLA, histology, and product contexts, they do not justify extrapolating activity across all sarcomas or engineered-cell platforms.

Individual clinical reports cannot, by themselves, describe how the wider development pipeline is organized. Basket trials can improve operational feasibility in rare cancers by evaluating a shared molecular or therapeutic feature across several histologies, but meaningful interpretation depends on prespecified disease strata, transparent denominators, and histology-specific reporting (23). Sarcoma-specific trials may offer clearer disease-level estimates yet often face slow accrual and reliance on multicenter or international cooperation, as illustrated by collaborative osteosarcoma research (24). Publications also tend to represent products that have progressed far enough to generate reportable clinical results, they provide limited visibility into planned, ongoing, inactive, terminated, or very early programs. Registry-level analysis can complement published evidence by mapping initiation over time, trial locations, development phase, status, sponsor, protocol-planned scale, disease scope, platform, and target. Such data still require cautious interpretation because registration and results-reporting fields may be incomplete, inconsistently structured, or difficult to use for comparative assessment (25). Moreover, a registered country indicates a trial-country link rather than actual patient access, enrolment, or referral capacity. A multidimensional record-level analysis is therefore needed to distinguish numerical growth from breadth, maturity, concentration, and reporting architecture.

The cellular-immunotherapy field is broader than receptor-engineered products. Tumor-infiltrating lymphocytes (TILs), dendritic-cell (DC) products, unmodified or cytokine-activated natural killer (NK) cells, and macrophage-directed approaches use distinct biological inputs, manufacturing workflows, and evidence standards. Combining these modalities in one quantitative denominator would conflate endogenous polyclonal recognition, antigen-presentation strategies, innate effector products, and genetically redirected receptor platforms. We therefore restricted the registry cohort to immune-cell products with explicit CAR or TCR engineering, for which the TrialTrove query and operational definition were reproducible. Adjacent cell-based approaches remain clinically relevant and are considered qualitatively in the Discussion, but they were not searched or counted as part of this cohort.

We conducted a TrialTrove-based registry analysis to characterize the clinical development landscape of CAR/TCR-engineered cellular therapies in musculoskeletal sarcomas. We examined temporal and geographic patterns, highest registered phase, registry status, sponsor, and protocol-planned scale, disease scope, platform, target, and reported adjunct strategies, and concentration and field availability. We evaluated one platform-classification edge case by excluding a single CAR/unspecified record and performed a complementary specified-antigen-only concentration analysis. Sarcoma-specific records were distinguished from broader solid-tumor protocols with explicit sarcoma eligibility. Eligibility was not interpreted as evidence of actual sarcoma enrollment, a dedicated sarcoma cohort, or a prespecified sarcoma analysis. The quantitative cohort was limited to products with explicit CAR or TCR receptor engineering and was not intended to inventory TIL, DC, unmodified NK, macrophage, or other non-CAR/TCR approaches.

## 2 Materials and methods

### 2.1 Study design and data source

We conducted a TrialTrove-based clinical trial landscape analysis. TrialTrove clinical trial data were retrieved on 10 June 2026, the proprietary export did not provide a separate exact database-cutoff timestamp. TrialTrove was the prespecified and sole data source for candidate identification, eligibility assessment, classification, and field extraction. ClinicalTrials.gov and other international registries were not searched or incorporated. The principal disease concepts were osteosarcoma, soft-tissue sarcoma, Ewing sarcoma, and sarcoma-related Medical Subject Headings, and the engineered-cell concepts were CAR-T, CAR-NK, TCR-T, chimeric antigen receptor, and T-cell receptor product categories. The complete TrialTrove expression is provided in **Supplementary Table 1**. A single-source design was used to preserve one search framework, data structure, and observation date. Expanding the study to multiple registries would require prospectively harmonized source-specific queries, aligned data snapshots, and cross-registry deduplication and was outside the prespecified protocol. The trial record, rather than the individual participant, was the primary unit of analysis. The study did not include patient-level efficacy or safety outcomes and was not designed as a therapeutic meta-analysis.

### 2.2 Eligibility assessment and record review

Eligible records described therapeutic interventional studies involving a musculoskeletal sarcoma population and an explicitly CAR/TCR-engineered immune-cell product. The disease scope included bone and soft-tissue sarcoma categories represented in the export. Broader solid-tumor basket records were retained only when TrialTrove-exported titles, disease labels, Medical Subject Headings, patient-population text, inclusion criteria, or trial objectives explicitly included sarcoma, these records formed a separate disease-scope stratum. Because the export lacked a standardized and consistently populated field for a dedicated or prespecified sarcoma cohort and did not provide histology-level actual accrual, we did not infer sarcoma enrollment or a sarcoma-specific expansion from eligibility alone. Eligible platforms comprised CAR-T, TCR-T, CAR-NK, TCR-NK, and other immune-cell products with explicit CAR or TCR engineering. Conventional systemic therapies, vaccines without a transferred engineered-cell product, unmodified immune cells, conventional TILs, DC or CIK/DC-CIK products, macrophage products without explicit CAR/TCR engineering, and other cellular products without explicit receptor engineering were excluded.

Two investigators independently reviewed all 140 candidate records for eligibility and independently assigned the standardized disease-scope, platform-family, recognition-target, and co-intervention classifications using prespecified rules. Ambiguous cases and disagreements were resolved by a third investigator. Review used fields contained in the TrialTrove export, including disease and product descriptors, intervention fields, titles, patient-population text, available eligibility text, trial objectives, and available identifiers. Each excluded record received one standardized reason. Of 140 candidates, 121 were included and 19 were excluded: 14 for non-target disease and five for non-target intervention. Potential duplicate representations were screened using exact TrialTrove IDs, non-blank NCT IDs, and normalized secondary protocol or registry identifiers. No repeated TrialTrove ID or non-blank NCT ID was found. Five groups of records shared a secondary identifier and were manually adjudicated by comparing sponsor protocol number, study title, sponsor, cellular product, target, registered phase, start date, participating countries, and other identifiers. All ten records were retained because they represented distinct sponsor protocols, sequential program studies, or separately registered master/substudy components rather than exact duplicate registrations. The final denominator therefore remained 121 records, adjudication details are provided in **Supplementary Table 2**.

### 2.3 Data extraction and variable standardization

Retained fields included TrialTrove and available NCT identifiers, protocol identifiers, title, disease terms, engineered-cell product and platform descriptors, target, registered phase and status, sponsor type, start date, country or region, protocol-planned enrollment, and reported co-interventions. Original fields were preserved alongside standardized variables. Excel serial dates were converted to calendar dates before start year was extracted. Phase was standardized as Phase I, I/II, II, or III and represented the highest registered phase. Sponsor type was grouped as Industry, Academic, Government, or Other/not reported. For integrated displays, Open and Planned were grouped as Open/planned, Completed, Closed, and Temporarily closed as Completed/closed, and Terminated remained separate. Planned enrollment was not interpreted as observed accrual. Missing values were retained and were not imputed.

### 2.4 Operational definitions

A sarcoma-specific record described a protocol directed to one or more sarcoma populations. A basket record explicitly including sarcoma described a broader solid-tumor protocol in which sarcoma eligibility was visible in TrialTrove-exported fields. This label does not establish actual sarcoma enrollment, a dedicated sarcoma cohort, or a prespecified sarcoma analysis. Each record was assigned one mutually exclusive disease category using a prespecified hierarchy: Basket, soft-tissue sarcoma, multiple sarcoma, or osteosarcoma. Single-platform CAR-T, TCR-T, and CAR-NK records were retained separately, TCR-NK, mixed-platform, and CAR/unspecified records were combined as Mixed/other in summary displays.

The recognition antigen was distinguished from cytokine payloads, armoring elements, marker genes, safety switches, and targets of conditioning or concomitant drugs. Target synonyms were standardized. The first antigen was treated as the primary target, and additional recognition antigens were retained as secondary targets. Records with no reported target or no single predefined antigen were represented as Unspecified in aggregate displays, this was a reporting category, not a biological target. Multi-target records were split and duplicate trial-target combinations removed to form deduplicated trial-target links. Target concentration was summarized both across all reporting links and after excluding Unspecified links to quantify the concentration of specified biological antigens. Reported countries were similarly split and deduplicated within records to form trial-country links.

Co-interventions were grouped as lymphodepletion, cytokine support, checkpoint blockade, radiation, vaccine/viral/RNA strategies, or other systemic/supportive strategies. Categories could overlap. A blank field was classified as not reported and was not interpreted as monotherapy. The CAR/unspecified CNK-UT record NCT05976906 remained in the primary cohort and was excluded only in the one-record platform-classification sensitivity analysis.

### 2.5 Statistical analysis and visualization

Categorical variables were summarized as counts and percentages, and protocol-planned enrollment as median and interquartile range (IQR). Field-specific analyses used available-case denominators, which are reported throughout. Annual and cumulative starts were calculated among records with a start year, 2026 was treated as a partial calendar year. Country analyses used deduplicated trial-country links, and target analyses used deduplicated trial-target links, neither represented individual participants or independent additional trials.

Open/planned proportions were accompanied by two-sided Wilson 95% confidence intervals (CIs). Sarcoma-specific and basket records were compared descriptively across eight prespecified characteristics. Differences were expressed in percentage points with Newcombe hybrid-score 95% CIs. Geographic concentration was described using Lorenz curves, Gini coefficients, and top-two-country shares. Target concentration was first described across all 125 deduplicated trial-target reporting links, including Unspecified as a reporting category, and then repeated after excluding the 12 Unspecified links, leaving 113 links to 22 specified antigens. Top-three shares and Gini coefficients were calculated for the overall, sarcoma-specific, and basket strata. These intervals and concentration metrics were descriptive, no comparative null-hypothesis testing or multiplicity-adjusted significance claims were made.

Alluvial and Marimekko diagrams summarized relationships among disease scope, sponsor, phase, status, platform, and primary target. UpSet displays summarized overlapping adjunct strategies and field-availability patterns. Target-strategy networks used deduplicated reported links, with edges shown at two or more links. The platform-classification sensitivity analysis repeated selected headline proportions after excluding the single CAR/unspecified record. It tested only this platform edge case and was not interpreted as robustness to disease-scope classification, target reporting, or trial-level uniqueness. The sarcoma-specific subset remained a scope comparison rather than an alternative primary cohort. Analyses were performed in Python 3.12 using pandas and NumPy, and figures were generated with Matplotlib 3.9.2.

## 3 Results

### 3.1 Study selection and final analytic cohort

Of 140 TrialTrove records subjected to prespecified eligibility assessment of disease scope and engineered-cell platform, 19 (13.6%) were excluded: 14 without an eligible sarcoma population and five without an eligible CAR/TCR-engineered cellular intervention. The primary analytic cohort therefore comprised 121 eligible TrialTrove records, including 38 sarcoma-specific records (31.4%) and 83 broader solid-tumor records with explicit sarcoma eligibility (68.6%) (**Figure 1**; **Table 1**). Review of five shared-secondary-identifier groups did not remove any record from the final denominator (**Supplementary Table 2**).

**Figure 1:**
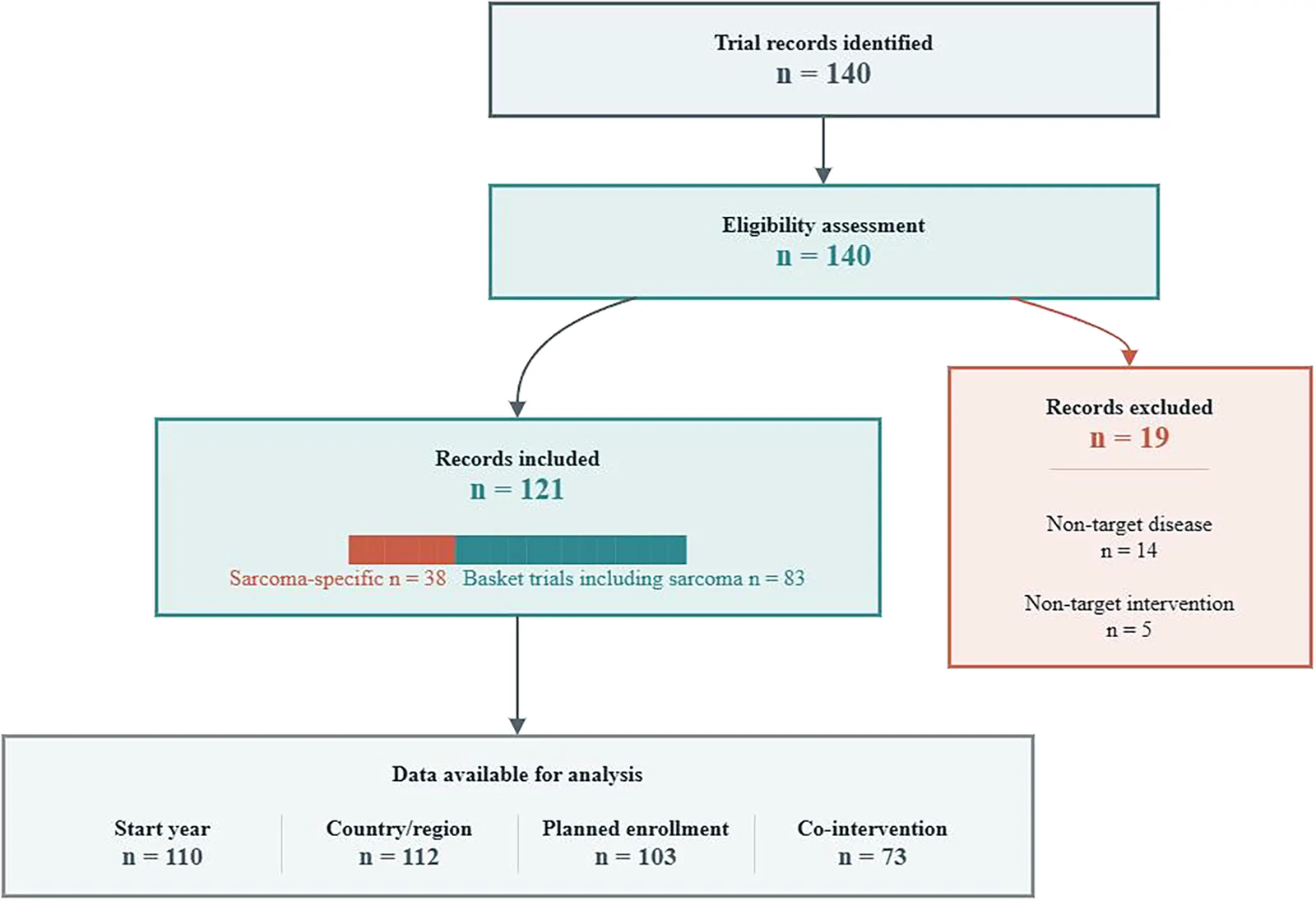
Screening flow and data availability. A total of 140 registry-derived trial records underwent prespecified eligibility assessment based on disease scope and engineered-cell platform. Nineteen records were excluded, including 14 without an eligible sarcoma population and five without an eligible CAR/TCR-engineered cellular intervention. The final analysis included 121 trial records: 38 sarcoma-specific records and 83 basket trial records explicitly including sarcoma. Available data comprised 110 records with a reported start year, 112 with country or region, 103 with protocol-planned enrollment, and 73 with a reported co-intervention. These availability denominators reflect registry-field reporting and are not additional screening exclusions.

**Table 1:** Characteristics of included clinical trials.

Section	Category	Overall	Sarcoma-specific	Basket including sarcoma
Disease scope	Sarcoma-specific	38 (31.4%)	38 (100.0%)	0 (0.0%)
	Basket including sarcoma	83 (68.6%)	0 (0.0%)	83 (100.0%)
Platform family	TCR-T	60 (49.6%)	19 (50.0%)	41 (49.4%)
	CAR-T	52 (43.0%)	19 (50.0%)	33 (39.8%)
	CAR-NK	5 (4.1%)	0 (0.0%)	5 (6.0%)
	Mixed/other	4 (3.3%)	0 (0.0%)	4 (4.8%)
Registered phase	Phase I	87 (71.9%)	22 (57.9%)	65 (78.3%)
	Phase I/II	21 (17.4%)	8 (21.1%)	13 (15.7%)
	Phase II	11 (9.1%)	6 (15.8%)	5 (6.0%)
	Phase III	2 (1.7%)	2 (5.3%)	0 (0.0%)
Registry status	Open	42 (34.7%)	10 (26.3%)	32 (38.6%)
	Planned	19 (15.7%)	7 (18.4%)	12 (14.5%)
	Completed	28 (23.1%)	12 (31.6%)	16 (19.3%)
	Closed/temporarily closed	11 (9.1%)	2 (5.3%)	9 (10.8%)
	Terminated	21 (17.4%)	7 (18.4%)	14 (16.9%)
Sponsor type	Industry	73 (60.3%)	27 (71.1%)	46 (55.4%)
	Academic	41 (33.9%)	9 (23.7%)	32 (38.6%)
	Government	5 (4.1%)	2 (5.3%)	3 (3.6%)
	Other/not reported	2 (1.7%)	0 (0.0%)	2 (2.4%)
Registered start period	2006-2015	10 (8.3%)	3 (7.9%)	7 (8.4%)
	2016-2020	37 (30.6%)	11 (28.9%)	26 (31.3%)
	2021-2026	63 (52.1%)	18 (47.4%)	45 (54.2%)
	Not reported	11 (9.1%)	6 (15.8%)	5 (6.0%)
Geographic scope	Single-country	97 (80.2%)	29 (76.3%)	68 (81.9%)
	Multicountry	15 (12.4%)	5 (13.2%)	10 (12.0%)
	Not reported	9 (7.4%)	4 (10.5%)	5 (6.0%)
Target specification	Specified target	109 (90.1%)	33 (86.8%)	76 (91.6%)
	Unspecified reporting category	12 (9.9%)	5 (13.2%)	7 (8.4%)
Planned enrollment	Reported, n/N (%)	103/121 (85.1%)	29/38 (76.3%)	74/83 (89.2%)
	Median (IQR), participants	24 (15-49)	20 (12-36)	24 (18-50)
	<25 participants, n (%)	57 (55.3%)	19 (65.5%)	38 (51.4%)
	25–49 participants, n (%)	20 (19.4%)	4 (13.8%)	16 (21.6%)
	>=50 participants, n (%)	26 (25.2%)	6 (20.7%)	20 (27.0%)

Data availability varied by field. A start year was available for 110 records (90.9%), country or region for 112 (92.6%), protocol-planned enrollment for 103 (85.1%), and a reported co-intervention for 73 (60.3%). An NCT identifier was available for 94 records (77.7%), and 109 (90.1%) had a specified primary target. These field-specific denominators were not additional screening exclusions.

### 3.2 Temporal evolution and geographic distribution

The 110 records with a start year spanned 2006–2026. Ten (9.1%) began in 2006–2015, 37 (33.6%) in 2016–2020, and 63 (57.3%) in 2021–2026. Annual starts remained at three or fewer through 2015, increased during 2016–2024, and reached 17 in 2025, the highest observed complete calendar year. Eleven additional records had a 2026 start year, 2026 was partial and was not compared directly with complete years (**Figure 2A**; **Table 1**). Platform composition shifted over time: eight of ten records dated 2006–2015 involved TCR-T, whereas 36 of 63 records dated 2021–2026 involved CAR-T and 20 involved TCR-T.

**Figure 2:**
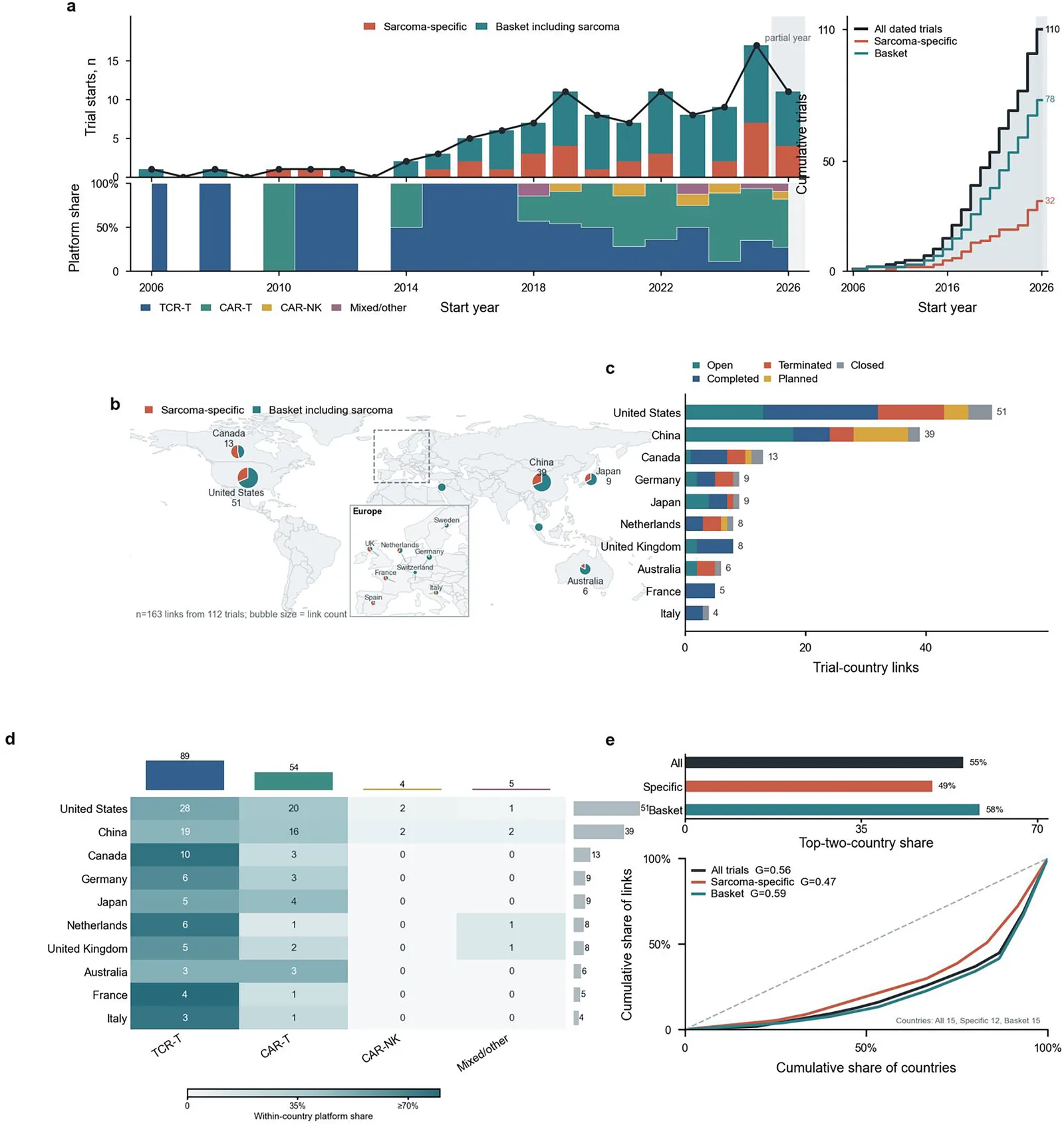
Temporal evolution and geographic distribution of CAR/TCR-engineered cellular therapy trials involving musculoskeletal sarcoma. **(a)**, Annual trial starts by disease scope among 110 trials with a reported start year, together with the total annual trajectory, cumulative trial counts, and annual platform composition, 2026 is a partial year. **(b)**, Global distribution among 112 trials with country information, represented by 163 trial-country links. Bubble size reflects the number of links, and sectors distinguish sarcoma-specific trials from basket trials including sarcoma, the inset enlarges Europe. **(c)**, Registry-status composition of the ten most represented countries. **(d)**, Country-platform matrix. Cell color indicates the within-country platform share, numbers show link counts, and marginal bars show platform and country totals. **(e)**, Geographic concentration expressed as the share contributed by the two most represented countries and Lorenz curves with Gini coefficients for the overall, sarcoma-specific, and basket-trial strata. Multi-country trials contribute once to each listed country. Registry status is a data-cutoff snapshot and is not interpreted as a clinical outcome.

The 112 records reporting location produced 163 deduplicated trial-country links across 15 countries. The United States contributed 51 links (31.3%), China 39 (23.9%), and Canada 13 (8.0%), Japan and Germany each contributed nine, and the United Kingdom and the Netherlands each contributed eight. The United States and China together contributed 55.2%. At the record level, 97 records were single-country, 15 multicountry, and nine had no country information. The overall country-link Gini coefficient was 0.560. The sarcoma-specific stratum contained 57 links, with a top-two-country share of 49.1% and Gini coefficient of 0.469, the basket stratum contained 106 links, with a top-two share of 58.5% and Gini coefficient of 0.590 (**Figures 2B–E**).

### 3.3 Clinical-development stage and protocol-planned scale

TCR-T accounted for 60 of 121 records (49.6%), CAR-T for 52 (43.0%), CAR-NK for five (4.1%), and Mixed/other platforms for four (3.3%). Phase I accounted for 87 records (71.9%), Phase I/II for 21 (17.4%), Phase II for 11 (9.1%), and Phase III for two (1.7%). Thus, 108 records (89.3%) were registered as Phase I or I/II. Phase II/III represented 8/38 sarcoma-specific records (21.1%) and 5/83 basket records (6.0%) (**Figures 3A, E**; **Table 1**).

**Figure 3:**
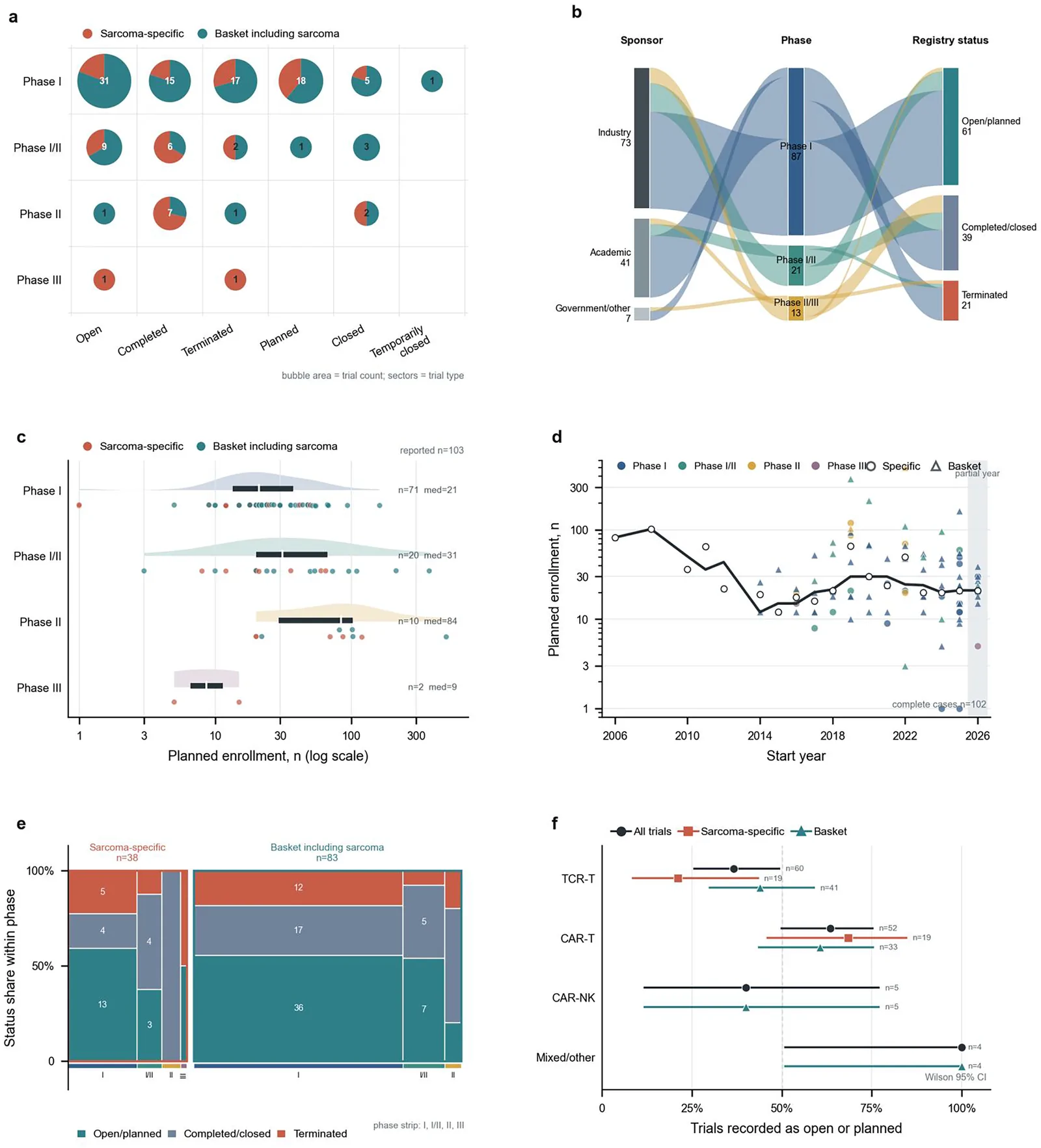
Clinical-development stage and planned trial scale of CAR/TCR-engineered cellular therapies involving musculoskeletal sarcoma. **(a)**, Phase-by-registry-status bubble matrix for all 121 included trials. Bubble area represents the number of trials, and sectors distinguish sarcoma-specific trials from basket trials including sarcoma. **(b)**, Alluvial representation linking sponsor class, development phase, and grouped registry status, flow width represents the number of trials. Government, other, and unreported sponsor classes were combined because of small counts, Phase II and III were grouped, and closed or temporarily closed trials were combined with completed trials for this descriptive flow. **(c)**, Planned-enrollment raincloud distributions by phase among 103 trials reporting this field. Half violins show the distribution, horizontal bars show the interquartile range with the median marked in white, and points represent individual trials colored by disease scope. **(d)**, Relationship between start year and planned enrollment among 102 complete cases. Colors denote phase, marker shapes denote disease scope, open circles show annual medians, the solid line shows the three-year pooled median, and 2026 is marked as a partial year. **(e)**, Marimekko plot integrating disease scope, phase, and grouped registry status. Main block width represents the number of trials in each disease-scope group, phase-block width represents the phase composition within that group, and vertical segmentation represents status composition within each phase, the lower strip identifies phase. **(f)**, Proportions of trials recorded as open or planned by platform and disease scope, with Wilson 95% confidence intervals and stratum denominators. Planned enrollment may differ from actual enrollment. Registry status is a data-cutoff snapshot and is not interpreted as clinical efficacy, failure, or maturity.

At the 10 June 2026 snapshot, 42 records were Open and 19 Planned, 28 were Completed, ten Closed, one Temporarily closed, and 21 Terminated. After grouping, 61 records (50.4%) were Open/planned, 39 (32.2%) Completed/closed, and 21 (17.4%) Terminated. Industry sponsors accounted for 73 records (60.3%), academic sponsors for 41 (33.9%), government sponsors for five (4.1%), and other or unreported sponsor types for two (1.7%) (**Figures 3A, B, E**).

Protocol-planned enrollment was reported for 103 records. The median was 24 participants (IQR 15–49), 57/103 records (55.3%) planned fewer than 25 participants, 20 (19.4%) planned 25–49, and 26 (25.2%) planned at least 50. Among sarcoma-specific records with data, the median was 20 (IQR 12–36, n=29), compared with 24 (IQR 18–50, n=74) among basket records. These values described planned sample size rather than observed accrual (**Figures 3C, D**; **Table 1**).

Planned enrollment increased across the principal early-phase categories: the median was 21 (IQR 13.5-37.5, n=71) in Phase I, 31.5 (IQR 20-67, n=20) in Phase I/II, and 84.5 (IQR 34-102.75, n=10) in Phase II. The two Phase III records were verified directly against the TrialTrove Trial Phase and Target Accrual fields: their planned enrollments were 5 and 15, yielding a median of 10. Phase was not inferred from sample size or reassigned during standardization. Start year and enrollment were jointly available for 102 records.

In the full cohort, 22/60 TCR-T records were Open/planned (36.7%, Wilson 95% CI 25.6%–49.3%), compared with 33/52 CAR-T records (63.5%, 49.9%–75.2%). The corresponding proportions within sarcoma-specific records were 4/19 and 13/19, and within basket records were 18/41 and 20/33. These intervals described registry status at the snapshot and were not comparative efficacy estimates (**Figure 3F**).

### 3.4 Disease-platform-target architecture and reported adjunct strategies

The record-level disease classification comprised 83 broader solid-tumor basket records with explicit sarcoma eligibility, 22 soft-tissue sarcoma records, 11 records involving multiple sarcoma categories, and five osteosarcoma records. The 83 basket records included 41 TCR-T, 33 CAR-T, five CAR-NK, and four Mixed/other records. The 38 sarcoma-specific records comprised 22 soft-tissue sarcoma, 11 multiple-sarcoma, and five osteosarcoma records (**Figure 4A**; **Table 1**). Basket-record counts indicate eligibility within a broader protocol and not necessarily actual sarcoma enrollment or a dedicated sarcoma expansion.

**Figure 4:**
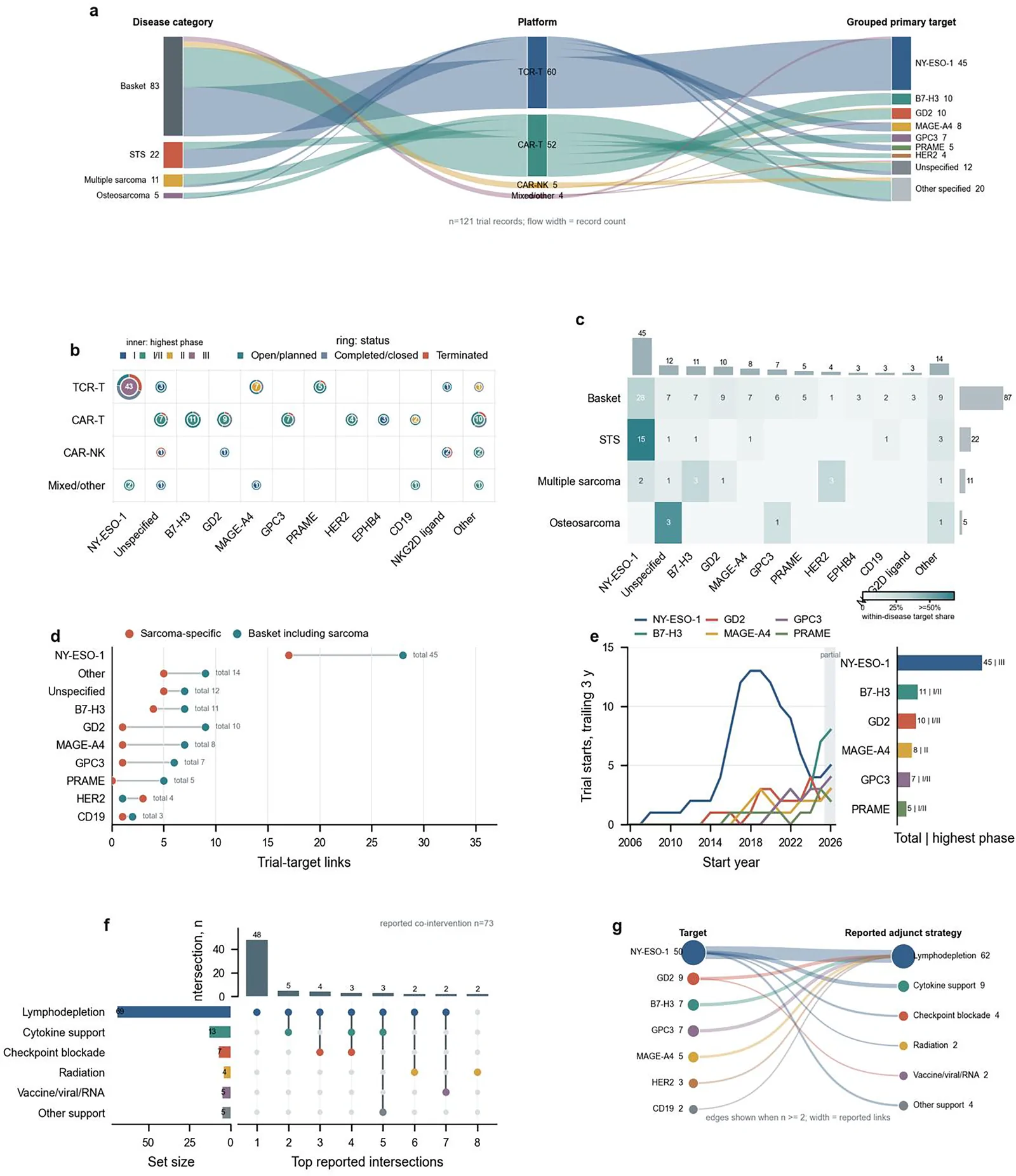
Disease, platform, target, and reported adjunct-strategy landscape of CAR/TCR-engineered cellular therapies involving musculoskeletal sarcoma. **(a)**, Alluvial representation linking disease category, platform family, and grouped primary target for all 121 included records. Each record contributes once, flow width represents record count. The seven most frequent specified primary targets are retained separately, while the remaining specified targets are grouped as Other specified. **(b)**, Platform-by-target matrix based on 125 deduplicated trial-target links after the three multi-target records were split into target components. Bubble area and the central label represent link count, inner-disc color represents the highest registered phase, and outer-ring sectors represent grouped registry-status composition. **(c)**, Disease-by-target heatmap using the same 125 links. Cell color represents the within-disease target share, cell labels show counts, and marginal bars show target and disease totals. **(d)**, Dual-lollipop comparison of sarcoma-specific and basket trial-target link counts for the ten most represented grouped targets. **(e)**, Temporal trial activity by target for the six most frequent specified targets. Lines show trailing three-year counts of newly started trials among 114 dated target links, the right strip shows the overall target-link total and highest registered phase. The 2026 interval is marked as a partial year. **(f)**, UpSet representation of standardized adjunct-strategy combinations among 73 records with a reported co-intervention. **(g)**, Bipartite target-strategy network, edges are displayed for at least two reported links. Missing co-intervention fields were not interpreted as monotherapy. Registry phase and status are descriptive snapshots and are not evidence of efficacy or clinical target utility.

After three multi-target records were split and duplicate within-record components removed, target analyses contained 125 deduplicated trial-target links. NY-ESO-1 contributed 45 links (36.0%), followed by Unspecified with 12 (9.6%), B7-H3 with 11 (8.8%), GD2 with ten (8.0%), MAGE-A4 with eight (6.4%), GPC3 with seven (5.6%), PRAME with five (4.0%), and HER2 with four (3.2%). NY-ESO-1 included 17 sarcoma-specific and 28 basket links. Of the 12 Unspecified links, 11 arose from records with no reported target and one from a record with no single predefined antigen, Unspecified was retained as a reporting category rather than a biological target (**Figures 4B–D**). Temporal trial activity for the six most frequent specified targets was summarized using trailing three-year counts of newly started trials (**Figure 4E**).

The largest disease-platform-target combinations were Basket–TCR-T–NY-ESO-1 (26 records) and soft-tissue sarcoma–TCR-T–NY-ESO-1 (15 records). The former comprised seven Open/planned, ten Completed/closed, and nine Terminated records, the latter comprised three, eight, and four, respectively. Other leading combinations included Basket–CAR-T–GD2 (eight), Basket–CAR-T–B7-H3 (seven), Basket–CAR-T–GPC3 (six), Basket–TCR-T–MAGE-A4 (six), and Basket–TCR-T–PRAME (five). The highest registered phase was Phase III for soft-tissue sarcoma–TCR-T–NY-ESO-1, Phase II for Basket–TCR-T–NY-ESO-1, and Phase I/II for the other listed combinations. These fields described development footprints rather than proof of clinical target utility (**Table 2**).

**Table 2:** Leading disease-platform-target combinations and development characteristics.

Disease category	Platform	Target category	Trials, n	Scope S/B, n	Highest phase	Status O/C/T, n	Median planned enrollment (reported n)
Basket	TCR-T	NY-ESO-1	26	0/26	Phase II	7/10/9	19 (n=22)
STS			15	15/0	Phase III	3/8/4	20 (n=10)
Basket	CAR-T	GD2	8	0/8	Phase I/II	4/4/0	48 (n=7)
		B7-H3	7	0/7		5/2/0	41 (n=7)
		GPC3	6	0/6		3/2/1	22 (n=6)
	TCR-T	MAGE-A4	6	0/6		4/2/0	21 (n=6)
		PRAME	5	0/5		4/0/1	24 (n=3)
	CAR-T	EPHB4	3	0/3	Phase I	3/0/0	33 (n=2)
		Unspecified (reporting category)	3	0/3	Phase I/II	3/0/0	54 (n=3)
Multiple sarcoma		B7-H3	3	3/0	Phase I	3/0/0	15 (n=3)
		HER2	3	3/0	Phase I/II	2/1/0	36 (n=3)
Basket	CAR-NK	NKG2D ligand	2	0/2	Phase I	1/0/1	18 (n=2)
	Mixed/other	NY-ESO-1	2	0/2	Phase I/II	2/0/0	64 (n=2)
	TCR-T	Unspecified (reporting category)	2	0/2	Phase I	2/0/0	21 (n=2)
Multiple sarcoma		NY-ESO-1	2	2/0		0/2/0	16 (n=2)
Osteosarcoma	CAR-T	Unspecified (reporting category)	2	2/0		0/1/1	21 (n=1)

Co-interventions were reported for 73 records and not reported for 48. Among records with data, lymphodepletion appeared in 69 (94.5%), cytokine support in 13 (17.8%), checkpoint blockade in seven (9.6%), vaccine/viral/RNA strategies in five (6.8%), other systemic/supportive strategies in five (6.8%), and radiation in four (5.5%), categories could overlap. Lymphodepletion alone was the largest reported intersection (48 records). The target-strategy network contained 106 reported links across 35 pairs, with NY-ESO-1–lymphodepletion the largest displayed pair (31 links). Records without co-intervention information were not classified as monotherapy (**Figures 4F, G**).

### 3.5 Structural gaps, field availability, and sensitivity analyses

Eight prespecified characteristics were compared descriptively between sarcoma-specific and basket records. The largest observed difference concerned Phase II/III representation: 21.1% versus 6.0%, a difference of 15.0 percentage points (Newcombe 95% CI 2.7–30.7). CAR-T represented 50.0% versus 39.8% (difference 10.2, 95% CI −8.3 to 28.3), Industry sponsorship 71.1% versus 55.4% (15.6, −3.2 to 31.7), and Open/planned status 44.7% versus 53.0% (−8.3, −26.2 to 10.6). Differences for recent start, planned enrollment of at least 50, and multicountry participation were smaller. No comparative hypothesis test was applied (**Figure 5A**).

**Figure 5:**
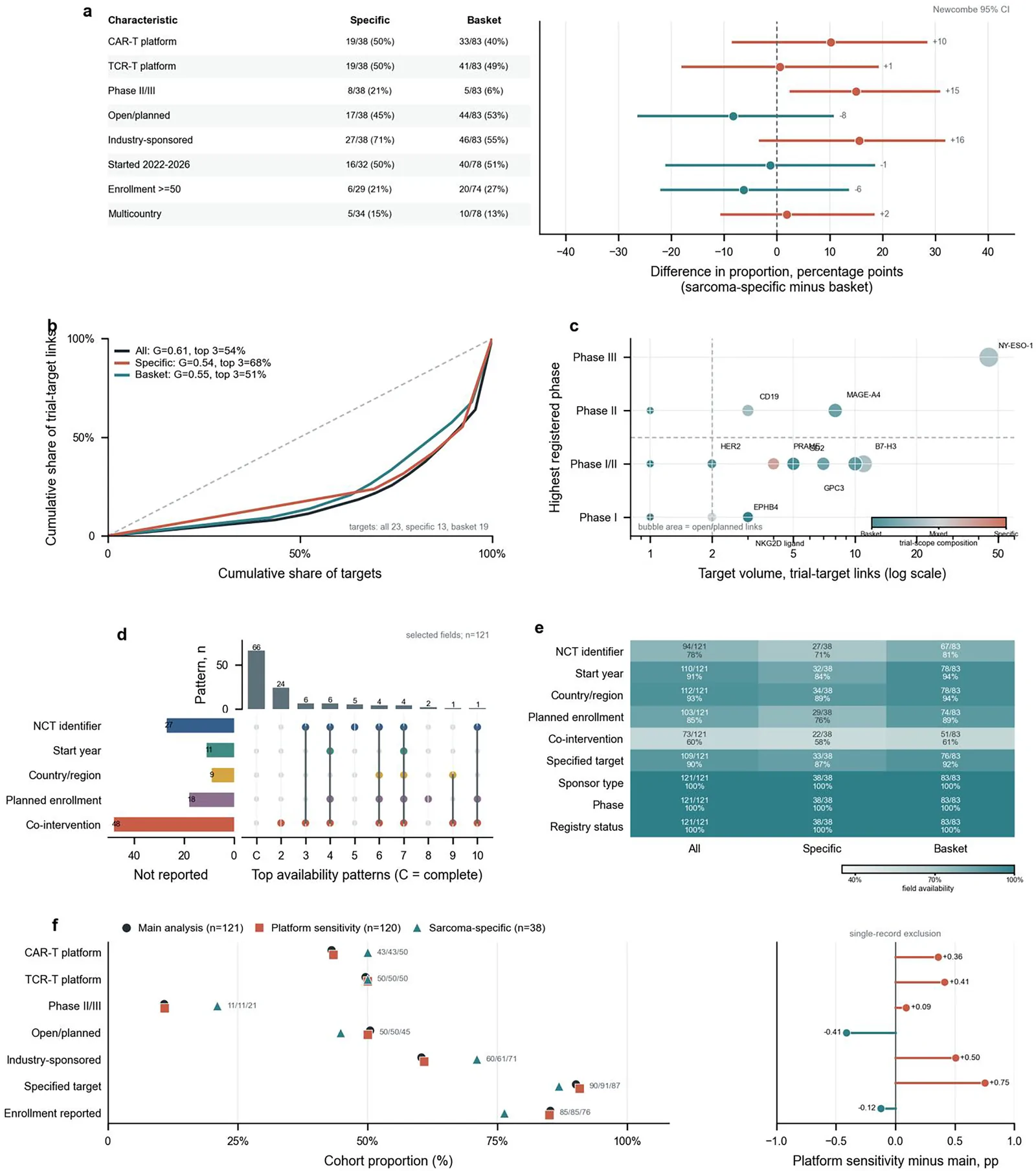
Structural gaps, reporting availability, and sensitivity analyses in the musculoskeletal-sarcoma cellular-therapy landscape. **(a)**, Difference-in-proportions forest plot comparing sarcoma-specific and basket records across eight observable characteristics, lines show Newcombe hybrid-score 95% CIs. **(b)**, Reporting-inclusive Lorenz curves of target concentration based on 125 deduplicated trial-target links, retaining Unspecified as a reporting category. **(c)**, Target-level relationship between portfolio volume and highest registered phase among 22 specified targets. **(d)**, UpSet representation of availability patterns for five selected fields among all 121 records. **(e)**, Field-availability matrix for the overall, sarcoma-specific, and basket strata. **(f)**, Platform-classification sensitivity profile comparing the main cohort with a 120-record set that excludes the single CAR/unspecified CNK-UT record, the right subpanel magnifies percentage-point changes after this one-record exclusion. This panel tests only that platform edge case and does not assess disease-scope, target-reporting, or uniqueness decisions. The complementary specified-antigen-only concentration analysis is reported in **Supplementary Table 3**. Registry phase and status are descriptive snapshots and are not efficacy or trial-quality outcomes.

Across all 125 trial-target reporting links, 23 specified-target or reporting categories were represented. The three largest categories contributed 68 links (54.4%), and the Gini coefficient was 0.615, this view retained Unspecified to describe registry reporting structure (**Figure 5B**). After the 12 Unspecified links were excluded, 113 links represented 22 specified antigens. NY-ESO-1 (45), B7-H3 (11), and GD2 (10) together contributed 66/113 links (58.4%), and the specified-antigen Gini coefficient was 0.624. In the sarcoma-specific stratum, the top-three specified-antigen share was 24/33 (72.7%, Gini 0.548), in the basket stratum it was 44/80 (55.0%, Gini 0.564). The specified-only analysis therefore strengthened, rather than created, the conclusion that the observed biological antigen portfolio was concentrated (**Supplementary Table 3**). The relationship between trial portfolio volume and the highest registered phase was descriptively examined across 22 specified targets (**Figure 5C**). Phase and volume were not treated as evidence of efficacy.

Field availability ranged from 60.3% for reported co-intervention to complete population of standardized sponsor, phase, and status. Sixty-six records (54.5%) were complete for the five selected fields in the missingness analysis, a further 24 lacked only a reported co-intervention (**Figures 5D, E**). The platform-classification sensitivity set excluded the single CAR/unspecified record and contained 120 records. Every displayed headline proportion changed by less than one percentage point (**Figure 5F**). This result applies only to that single platform-classification edge case, the specified-antigen-only analysis addressed target reporting separately (**Supplementary Table 3**).

## 4 Discussion

This TrialTrove-based registry analysis shows that clinical development of CAR/TCR-engineered cellular therapies involving musculoskeletal sarcomas has expanded substantially, but the observed portfolio is not yet broadly distributed or mature. More than half of dated records began during 2021-2026, almost nine in ten remained at Phase I or I/II, and more than half of records with planned enrollment proposed fewer than 25 participants. Broader solid-tumor protocols with explicit sarcoma eligibility outnumbered sarcoma-specific records by more than two to one, but eligibility did not establish actual sarcoma enrollment or a dedicated cohort. Development was also concentrated geographically and technologically: the United States and China contributed more than half of trial-country links, TCR-T and CAR-T accounted for most records, and NY-ESO-1 dominated both the reporting-inclusive and specified-antigen views. These findings describe TrialTrove records, not treatment effects, product superiority, or all cell-therapy approaches.

The temporal pattern adds nuance to the overall growth signal. Early development was dominated by TCR-T records, whereas CAR-T accounted for more than half of records beginning in 2021–2026. This shift may reflect expanding surface-antigen programs, broader manufacturing experience, and increased interest in pediatric and adult solid tumors, but the registry analysis cannot assign causes. Nor does a rising count necessarily indicate an equivalent rise in independent products, treated participants, or mature evidence: related programs may appear across phases, targets can recur in multiple basket protocols, and start year is a registered or planned field rather than a verified activation date. The peak of 17 starts in 2025 is therefore best interpreted as a portfolio-activity marker. The 2026 count was deliberately labelled partial, preventing a visually convenient but misleading comparison between an incomplete year and prior complete years.

The predominance of basket records is understandable in rare and heterogeneous diseases, because shared screening, manufacturing, and operational infrastructure may make target-defined studies feasible when stand-alone accrual is slow. The same efficiency creates an interpretive constraint. In this analysis, the basket label required explicit sarcoma eligibility, but TrialTrove did not provide a standardized, consistently populated field for a dedicated sarcoma cohort, prespecified sarcoma analysis, or histology-level actual accrual. Accordingly, the 83 basket records define an eligibility footprint and should not be read as 83 protocols that necessarily enrolled sarcoma patients or pursued sarcoma-directed clinical development. Methodological studies show that cross-basket information borrowing can improve efficiency, but its validity depends on heterogeneity, subgroup prevalence, and prespecified calibration (26, 27). Future trials should preserve independently analyzable sarcoma cohorts, report screened, enrolled, and treated histology-specific denominators, and justify any cross-histology borrowing biologically and statistically.

The early-phase and small-scale profile should likewise be interpreted as a feature of the current development stage, not as evidence of failure. First-in-human engineered-cell studies must address manufacturing feasibility, conditioning, dose, acute toxicity, trafficking, expansion, and persistence before comparative efficacy questions can be considered. These requirements are especially demanding in rare sarcomas, in which target expression, HLA eligibility, previous therapy, age, and referral to specialized centers can narrow the available population. The median protocol-planned enrollment of 24 and the predominance of Phase I records therefore reflect both biological uncertainty and operational constraints. Multicenter collaboration may enlarge eligible populations, but it also requires sustained regulatory, data, safety, and funding infrastructure. Registry status should be read cautiously. Open or planned status denotes portfolio activity at a snapshot, while completion, closure, or termination can arise for operational, strategic, safety, biological, or administrative reasons. Without linked patient-level results, none of these categories supports an inference about efficacy or futility.

The platform and target architecture reveals both accumulated experience and portfolio dependence. TCR-T was the largest platform family overall, and NY-ESO-1 contributed more than one-third of target links. These counts establish where development has concentrated, not whether target expression was adequate in enrolled tumors or whether a product produced benefit. For HLA-restricted products, target naming alone is insufficient because loss or downregulation of major histocompatibility complex molecules can provide a route of immune escape (28). Evidence from advanced pediatric solid cancers, including sarcomas, further shows heterogeneous HLA expression, recurrent HLA loss of heterozygosity, and transcriptional silencing affecting HLA-B and antigen-processing machinery (29). Future TCR-T records and publications would therefore be more informative if they linked HLA genotype, tumor antigen expression, antigen-processing competence, and longitudinal resistance assessment to subtype-specific denominators. These variables were not measured in the present registry analysis, so their relevance to individual records remains a biological and design consideration rather than an observed explanation.

CAR programs address cell-surface antigens without HLA restriction, but the registry portfolio remains exploratory. In the reporting-inclusive view, Unspecified was the second-largest category, because it is not a biological antigen, we repeated concentration analyses after excluding its 12 links. The specified-only analysis showed that NY-ESO-1, B7-H3, and GD2 accounted for 58.4% of 113 specified-antigen links and yielded a Gini coefficient of 0.624, confirming substantial biological target concentration. These counts identify development hypotheses rather than comparable expression, safety, or activity across sarcoma subtypes. Antigen heterogeneity or loss, limited trafficking, and insufficient persistence remain recurring barriers in solid-tumor CAR-T development (30). Diversification should therefore be guided by reproducible tumor expression, spatial sampling, normal-tissue safety, mechanistic evidence, and feasible patient-selection assays rather than by target novelty alone.

Conditioning and adjunct strategies form another part of the product context. Lymphodepletion was reported in nearly all records that contained a co-intervention field, while cytokine support, checkpoint blockade, radiation, and vaccine, viral, or RNA strategies appeared much less often and in overlapping combinations. A systematic review of solid-tumor TCR-T studies found heterogeneous lymphodepletion regimens, limited comparative evidence, and gaps in toxicity reporting, illustrating why the transferred cells and their conditioning cannot be interpreted separately (31). Our counts cannot compare regimen effectiveness because reporting was incomplete, categories were non-exclusive, and the dataset did not contain dose, timing, adherence, or patient outcomes. Protocols and publications should therefore specify the complete therapeutic package, distinguish mandatory conditioning from experimental combinations, and link each component to safety and cellular-kinetic analyses. This would help separate limitations attributable to the cell product from those associated with host preparation or other modifiable treatment conditions.

Geographic concentration has scientific and operational implications. The United States and China together contributed 55.2% of trial-country links, and basket records were more geographically concentrated than sarcoma-specific records by the descriptive measures used here. These findings identify where registered development activity was located, but they do not show how many patients were screened, enrolled, treated, or able to access care. A country link may represent one site or many. Research on US cancer trials shows that site distribution, travel burden, and social vulnerability can remain unequal within a country, supporting a clear distinction between geographic presence and patient access (32). Engineered-cell products add manufacturing slots, turnaround time, product quality, transport, reimbursement, and post-infusion monitoring to that access pathway (33). Early development also depends on scalable good-manufacturing-practice workflows, assay development, and quality control (34). Multicenter and cross-border networks may increase the eligible population and distribute expertise, but they require harmonized testing, logistics, regulation, and toxicity management. Future landscape studies should therefore distinguish registration geography from site density, patient access, and manufacturing reach.

Field visibility was another structural limitation of the observed landscape. Start year, country, target, and planned enrollment were available for most, but not all, records, whereas a reported co-intervention was available in only 60.3%. Treating an empty adjunct field as monotherapy would consequently create a false treatment category. More broadly, trial-database records are dynamic documents designed for prospective transparency and trial identification, not complete substitutes for protocols, statistical analysis plans, or publications. Recent work on registry-entry generation found that structured elements could be populated more consistently than some narrative fields, supporting field-level rather than all-or-nothing interpretations of completeness (35). A separate audit of a public trial registry identified incomplete and variable data availability, illustrating a general limitation of registry-derived research rather than a deficiency measured directly in TrialTrove (36). We preserved original TrialTrove fields, used available-case denominators, and separated record and link units. These precautions improve reproducibility, but they cannot restore information that was absent from the export or capture updates after the snapshot.

### 4.1 Adjacent cell-based strategies

CAR/TCR-engineered products are only one component of the sarcoma cell-therapy landscape. TIL therapy can preserve polyclonal recognition of patient-specific antigens and has shown feasibility in a small metastatic soft-tissue sarcoma pilot, but it depends on tumor procurement, successful TIL expansion, product potency, and control of a suppressive microenvironment, the available sarcoma evidence remains limited (40). DC-based strategies can present multiple autologous tumor antigens and may be combined with effector-cell transfer or checkpoint modulation, yet a phase I/II study of tumor-lysate-pulsed DCs in refractory bone and soft-tissue sarcoma reported acceptable safety but clinical benefit in only a small minority (41). Unmodified or expanded NK cells offer HLA-unrestricted innate cytotoxicity and potential donor-derived availability, but expansion, homing, persistence, and tumor-microenvironment suppression remain barriers. Macrophage-directed approaches, including CAR macrophages (CAR-M), may exploit tumor infiltration, phagocytosis, antigen presentation, and microenvironment remodeling, but the supporting solid-tumor evidence is still predominantly preclinical and manufacturing, phenotypic plasticity, and control of inflammatory activity remain unresolved (42). These approaches may ultimately complement engineered T- and NK-cell products, but their distinct mechanisms and development pathways justify qualitative comparison rather than retrospective inclusion in the present quantitative denominator.

This study has several strengths. Two investigators independently screened records and assigned standardized classifications, with a third investigator adjudicating disagreements. Potential duplicate representations were assessed using exact and normalized identifiers plus study-level characteristics, and the five shared-secondary-identifier groups are documented transparently. The analysis separated sarcoma-specific from basket eligibility records, trial records from country and target links, and Unspecified reporting from specified biological antigens. Descriptive interval estimates, a specified-antigen-only analysis, and a narrowly defined one-record platform-classification sensitivity analysis made key assumptions visible without unsupported significance or efficacy claims.

The limitations are equally important. First, TrialTrove is a proprietary commercial database, and records absent from its export may have been missed, ClinicalTrials.gov and other registries were not searched or incorporated. Second, the 10 June 2026 export did not provide a separate exact database-cutoff timestamp, and later record versions may differ. Third, manual standardization and uniqueness adjudication reduce but cannot eliminate uncertainty. A shared identifier can represent a duplicate registration, a sequential program, or linked master and substudy components, our unit is therefore the adjudicated TrialTrove record rather than a claim that every record is a wholly independent underlying clinical study. Fourth, the export did not reliably identify dedicated sarcoma cohorts within basket protocols or actual histology-level accrual. Its broad disease and Medical Subject Heading labels also did not support a reproducible histology-frequency table without risking false precision. Fifth, the one-record platform-classification sensitivity analysis tested only that platform edge case and does not demonstrate robustness to disease-scope classification, target reporting, or record uniqueness. Finally, no patient-level screening, treatment, safety, response, survival, or manufacturing data were available, planned enrollment may differ from actual accrual, highest registered phase is not a maturity score, country links do not measure access, and missing fields constrained some analyses.

### 4.2 Engineering strategies and future clinical development

The observed early-stage, small-scale portfolio makes product definition and manufacturing reporting especially important. CAR binding, hinge, signaling, and costimulatory modules and TCR affinity, HLA restriction, chain pairing, and cross-reactivity can influence activity and risk (30). Receptor transfer by viral vectors, transposons, RNA, or targeted editing also changes expression kinetics and release testing. Trials should report collection-to-release time, manufacturing failure, dose yield, product comparability, normal-tissue antigen testing, cellular kinetics, and long-term safety. Donor-derived or universal products may improve availability, but off-target editing, alloreactivity, rejection, and cytopenia require direct sarcoma evaluation rather than extrapolation from hematological malignancies (43).

The target concentration and incomplete adjunct-strategy reporting also define priorities for next-generation studies. Multi-antigen logic, armoring, trafficking modifications, and gene-edited allogeneic products may address escape, suppression, or access, but remain hypotheses until tested in sarcoma with prespecified safety controls (37, 44). Combinations with checkpoint blockade, radiotherapy, vaccines or oncolytic approaches, and microenvironment-directed agents should be tied to a mechanism and accompanied by correlative sampling. Dose, sequence, timing, lymphodepletion, overlapping toxicity, and the complete product context should be reported, registry counts cannot determine whether a combination adds benefit.

The portfolio supports an evidence-calibrated development hierarchy rather than a numerical ranking. Biomarker-selected TCR-T programs targeting MAGE-A4 or NY-ESO-1 in defined HLA and histology contexts have the most mature human precedent, although HLA eligibility, antigen expression, and processing constrain applicability. B7-H3-, GD2-, and HER2-directed CAR programs remain promising but require subtype-specific safety, activity, and resistance data, CAR-NK, CAR-M, and universal products are earlier-stage. Efficient translation will require sarcoma-specific cohorts or independently analyzable basket expansions, prospective antigen/HLA enrichment, adaptive stopping and expansion, justified information borrowing, longitudinal tumor sampling, cellular kinetics, manufacturing endpoints, and harmonized safety, response, durability, and resistance definitions (38, 39).

## 5 Conclusion

The clinical development landscape of CAR/TCR-engineered cellular therapies involving musculoskeletal sarcomas has expanded rapidly, particularly since 2021, but remains dominated by early-phase and small planned studies, broader solid-tumor protocols listing sarcoma eligibility, a limited number of countries, and a concentrated specified-antigen portfolio. Basket eligibility should not be interpreted as actual sarcoma enrollment or a dedicated sarcoma cohort. Future trials should preserve independently analyzable sarcoma cohorts, integrate antigen/HLA selection and longitudinal resistance sampling, and harmonize reporting of conditioning, adjunct strategies, cellular kinetics, manufacturing feasibility, safety, response, and durability. TIL, DC, unmodified NK, macrophage, and other non-CAR/TCR approaches remain relevant but were outside this quantitative cohort. Because the analysis used adjudicated TrialTrove records without patient-level outcomes, it should not be interpreted as evidence of comparative efficacy or a clinical recommendation for any platform or antigen.

## Data Availability

The data analyzed in this study were obtained from TrialTrove, a proprietary clinical trial database. Access to the underlying data is subject to the database provider's licensing terms. Derived data supporting the findings may be made available by the corresponding author upon reasonable request, subject to applicable data-sharing restrictions.
