# Supplementary material for: Clinical development landscape of CAR/TCR-engineered cellular therapies in musculoskeletal sarcomas: a TrialTrove-based registry analysis
Source: Front Immunol. 2026 Oct 28;17:1939952. doi: 10.3389/fimmu.2026.1939952 (PMC13752143; doi:10.3389/fimmu.2026.1939952)
Supplement: Supplementary file 1 [file DataSheet1.pdf]

Supplementary Table S1. Search strategy, eligibility criteria, and operational definitions

Domain	Item	Operational definition
	Database	TrialTrove clinical trial export.
Data source	Data date and source boundary	TrialTrove clinical trial data were retrieved on 10 June 2026. TrialTrove was the prespecified and sole source used for candidate identification, eligibility assessment, classification, and field extraction. ClinicalTrials.gov and other international registries were not searched or incorporated.
Data source	External-registry scope	ClinicalTrials.gov and other international registries were not incorporated. A multi-registry extension would require prospectively harmonized source-specific queries, aligned data snapshots, and cross-registry deduplication and was outside the prespecified single-source design.
Search strategy	Principal disease concepts	Osteosarcoma and soft-tissue sarcoma disease fields, together with Medical Subject Headings for Ewing sarcoma, osteosarcoma, sarcoma, and soft-tissue sarcoma.
Search strategy	Principal engineered-cell concepts	CAR-NK, TCR-T, chimeric antigen receptor, T-cell receptor, and CAR-T TrialTrove cell-technology product categories.
Record handling	Duplicate checking	Exact TrialTrove and non-blank NCT identifiers were checked first. Five groups sharing a normalized secondary identifier were then adjudicated using sponsor protocol number, title, sponsor, product, target, phase, start date, countries, and other identifiers. No record was removed; see Supplementary Table S2.
Search strategy	Exact TrialTrove expression	[[(Disease is Oncology: Osteosarcoma) OR (Disease is Oncology: Soft Tissue Sarcoma)] OR [(MeSH Term is Ewing Sarcoma) OR (MeSH Term is Osteosarcoma) OR (MeSH Term is Sarcoma) OR (MeSH Term is Soft Tissue Sarcoma)]] AND [(Drug Type is Biological > Cellular > Cell technology type > Chimaeric antigen receptor > CAR-NK cell) OR (Drug Type is Biological > Cellular > Cell technology type > T cell receptor > TCR-T cell) OR (Drug Type is Biological > Cellular > Cell technology type > Chimaeric antigen receptor) OR (Drug Type is Biological > Cellular > Cell technology type > T cell receptor) OR (Drug Type is Biological > Cellular > Cell technology type > Chimaeric antigen receptor > CAR-T cell)]
Eligibility	Disease scope	Osteosarcoma, Ewing sarcoma, soft-tissue sarcoma, synovial sarcoma, rhabdomyosarcoma, chondrosarcoma, chordoma, malignant peripheral nerve sheath tumour, and other musculoskeletal sarcoma categories represented in the export.
	Basket trials	Broader solid-tumour records were included only when exported fields explicitly showed sarcoma eligibility. The export did not permit consistent classification of dedicated sarcoma cohorts, prespecified sarcoma analyses, or actual sarcoma enrollment; eligibility was not interpreted as evidence of these features.
	Eligible engineered-cell platforms	CAR-T, TCR-T, CAR-NK, TCR-NK, and other clearly CAR/TCR-engineered immune-cell therapies.
	Excluded interventions	Conventional checkpoint inhibitors, chemotherapy, targeted drugs, antibody-drug conjugates, vaccines, unmodified NK cells, conventional tumour-infiltrating lymphocytes, CIK/DC-CIK products, and cellular products without explicit CAR/TCR receptor engineering.
	Screening result	Two investigators independently assessed all 140 candidates and assigned standardized classifications; a third investigator adjudicated disagreements. A total of 121 records were included and 19 excluded (14 non-target disease; five non-target intervention).
Operational definition	Trial scope	Sarcoma-specific indicates a protocol directed to sarcoma populations; Basket including sarcoma indicates a broader solid-tumour protocol with explicit sarcoma eligibility.
	Disease category	One analysis category per trial record: Basket, soft-tissue sarcoma, multiple sarcoma, or osteosarcoma, using the prespecified v3 hierarchy.
	Platform family	Single-platform CAR-T, TCR-T, and CAR-NK records were retained separately; mixed platforms, TCR-NK combinations, and CAR/unspecified products were grouped as Mixed/other in summary analyses.
	Target	Target synonyms were standardized and multi-target records split into deduplicated trial-target links. Unspecified was retained only for reporting-structure displays and excluded in a complementary specified-antigen-only concentration analysis.
	Registered phase	Phase I, Phase I/II, Phase II, or Phase III as recorded after v3 standardization. Highest registered phase is descriptive and is not evidence of efficacy or clinical target utility.
	Registry status	Open and Planned were grouped as Open/planned; Completed, Closed, and Temporarily closed were grouped as Completed/closed; Terminated was retained separately. Status is a registry snapshot, not an outcome.
	Sponsor type	Industry, Academic, Government, and Other/not reported. The combined final category prevents a single unclassified sponsor type from being interpreted as a distinct sponsor sector.
	Registered start year	The registry-reported or planned start date was converted from Excel serial values to an ISO date and then to year. It should not be interpreted solely as an observed activation date.
	Country and geographic scope	A trial was multicountry when more than one country/region was listed. Country analyses use deduplicated trial-country links, so one multicountry trial contributes once to each listed country.
	Planned enrollment	Protocol-planned sample size; no missing value was imputed and planned enrollment was not interpreted as actual accrual.

Domain	Item	Operational definition
	Reported adjunct strategy	Reported co-interventions were standardized as lymphodepletion, cytokine support, checkpoint blockade, radiation, vaccine/viral/RNA, or other systemic/support strategies. Blank fields were labelled not reported rather than monotherapy.
Missing-data rule	Panel-specific denominators	Start year, country/region, planned enrollment, and co-intervention analyses used available records only. Denominators were reported for every analysis and no missing value was imputed.
Analysis unit	Counts	The primary unit is the trial record. Target analyses use deduplicated trial-target links and geographic analyses use deduplicated trial-country links; neither represents patients.
Sensitivity analysis	Platform-classification edge case	NCT05976906 was retained in the main cohort and excluded in a one-record platform-classification sensitivity analysis. This did not test disease-scope, target-reporting, or uniqueness decisions.

Note 1. Rules were applied to the locked v3 dataset; missing dates, countries, enrollment, and co-interventions were not imputed.

Supplementary Table S2. Adjudication of records sharing normalized secondary identifiers

Shared identifier	TrialTrove records	Study-level comparison	Adjudication
EudraCT 2019-004446-14	382773 (NCT04526509); 477408 (NCT05943990)	Protocol 209012 master record versus 209012 Substudy 2; master product set included GSK3901961 and GSK3845097, whereas the substudy record focused on GSK3845097; titles and NCT identifiers differed, while sponsor, start date, and countries overlapped.	Retained as separately registered master/substudy protocol components rather than exact duplicate registrations; linkage is acknowledged.
HEROS 3.0	127585 (NCT00902044); 410669 (NCT04995003)	Sponsor protocols 0903-969 and H-49271; starts in 2010 and 2021; Phase I/II and Phase I; later study added checkpoint blockade.	Retained as distinct sequential HER2 CAR-T protocols sharing a programme label.
ACTengine	293909 (NCT03247309); 333619 (NCT03686124)	IMA201/MAGE-A4 versus IMA203/PRAME; different sponsor protocol numbers, NCT identifiers, registered phases, and start dates.	Retained as distinct product-specific protocols sharing a programme label.
ChiCTR-OIC-17013872	301913 (NCT03159585); 320169 (NCT03462316)	Sponsor protocols 2017-ZLZX-001 and B2017-023-01; broader solid-tumour versus bone/soft-tissue-sarcoma titles; starts in 2017 and 2018; same TAEST16001 product programme.	Retained as distinct sponsor protocols within a related product programme.
JapicCTI-152896	252472 (NCT02366546); 284521 (NCT02869217)	Sponsor protocols 1301-01 and 1301-02; Phase I and Phase Ib/standardized Phase I; starts in 2015 and 2016; titles and participating-country fields differed.	Retained as distinct sequential protocols within the same product programme.

Note. Adjudication was performed on the TrialTrove export. The 121-record denominator was unchanged. The master/substudy relationship is retained transparently as separately registered protocol components and should not be interpreted as proof of fully independent underlying clinical studies.

Supplementary Table S3. Specified-antigen-only target-concentration analysis

Stratum	All / specified links	Specified antigens, n	Top three specified antigens (links)	Top-three share; Gini
Overall	125 / 113	22	NY-ESO-1 (45); B7-H3 (11); GD2 (10)	66/113 (58.4%); 0.624
Sarcoma-specific	38 / 33	12	NY-ESO-1 (17); B7-H3 (4); HER2 (3)	24/33 (72.7%); 0.548
Basket including sarcoma	87 / 80	18	NY-ESO-1 (28); GD2 (9); MAGE-A4 (7)	44/80 (55.0%); 0.564

Note. Specified-only analyses excluded 12 Unspecified reporting links: five from sarcoma-specific records and seven from basket records. Multi-target records contributed one deduplicated link per recognition antigen. Unspecified was excluded because it is a reporting category rather than a biological antigen.
